# Sperm competition dynamics: ejaculate fertilising efficiency changes differentially with time

**DOI:** 10.1186/1471-2148-8-332

**Published:** 2008-12-16

**Authors:** Tommaso Pizzari, Kirsty Worley, Terry Burke, David P Froman

**Affiliations:** 1 Edward Grey Institute, Department of Zoology, University of Oxford, Oxford, OX1 3PS, UK; 2 School of Biological Sciences, University of East Anglia, Norwich NR4 7TJ, UK; 3 Sheffield Molecular Genetics Facility, Department of Animal & Plant Sciences, University of Sheffield, Sheffield, S10 2TN, UK; 4 Department of Animal Sciences, Oregon State University, 112 Withycombe Hall, Corvallis, OR 97331 USA

## Abstract

**Background:**

A fundamental challenge in evolutionary biology is to resolve the mechanisms that maintain paternity a hypervariable fitness component. Because females are often sexually promiscuous, this challenge hinges on establishing the mechanisms through which the ejaculates of different males compete for fertilisation (sperm competition). The competitive quality of an ejaculate is mediated by the relative number of live sperm and their motile performance. The differential rate at which rival ejaculates lose their fertilising efficiency over time is therefore expected to influence the outcome of sperm competition.

**Results:**

Here, we artificially inseminated into sets of replicate domestic hens, *Gallus gallus domesticus*, experimentally engineered heterospermic ejaculates containing a large number of low-quality sperm from one male, and a lower number of high-quality sperm from another male. Large, low-quality ejaculates fertilised the first eggs produced after insemination, but small, high-quality ejaculates prevailed in the long run despite their numerical disadvantage.

**Conclusion:**

Together, these results provide the first experimental demonstration that the relative competitive value of an ejaculate changes drastically over the time during which competing ejaculates are stored within the reproductive tract of a female, resulting in a marked temporal pattern of variation in paternity. A high level of replication makes these results robust. However, our study was restricted to few males of a well characterised study population, and future work should explore the generality of these results.

## Background

Male fertilisation success (paternity) is often extremely variable within populations, and an enduring fundamental challenge in evolutionary biology is to understand the mechanisms that explain and maintain such variability in the face of Darwinian selection [1]. In the majority of sexually reproducing species, the ejaculates of different males can compete to fertilise the eggs of a female, a process called sperm competition, and recent evidence indicates that a large source of variation in paternity can be determined by sperm competition dynamics after insemination [2-10]. Two general ejaculate traits influence fertilisation success under sperm competition [11]: the relative number of sperm delivered by competing ejaculates (ejaculate size) [12,13], and the motile performance of inseminated sperm (ejaculate quality) [14-16]. However, the mechanisms through which ejaculate size and quality contribute to determine fertilisation success under sperm competition remain poorly understood. This problem is particularly pronounced in internally-fertilising species, where rival ejaculates co-occur in the reproductive tract of a female, making sperm competition both prolonged and difficult to study. Here, the probability that an ejaculate achieves fertilisation on a given point in time following insemination depends on the rate at which its fertilising efficiency declines relative to rival ejaculates [17-20]. However, the prevailing experimental approach has been to study the extent to which the characteristics of rival ejaculates, such as size and quality, measured at ejaculation, predict the share in paternity of all of the offspring produced by the inseminated female over a given period of time following insemination. Such an overall measure of reproductive success fails to reveal time-dependent variation in the relative fertilising efficiency of competing ejaculates. Here, we experimentally study the rate at which rival ejaculates of varying size and quality accumulate fertilisation success over sperm storage time in an avian model system, the domestic fowl, *Gallus gallus domesticus*.

Female birds store sperm in specialised sperm-storage organs, the Sperm Storage Tubules (SSTs). Sperm are continuously lost from the SSTs and move to the infundibulum, where fertilisation occurs. The continuous output of sperm from the SSTs translates into an advantage of the ejaculate that is numerically most represented in the SSTs [11]. Ejaculate size and quality are known to influence the overall outcome of sperm competition in birds [21-23]. However, female birds store viable sperm for a prolonged period of time that ranges from few days in some species to up to four weeks in others [24,25]. The mechanisms that regulate sperm storage in the SSTs and loss from the SSTs over time are part of the solution to an enduring puzzle that has eluded reproductive biologists for over 70 years [23,24,26,27], and consequently the dynamics of sperm competition have remained unresolved.

Previous studies using artificial inseminations indicated that both ejaculate size and quality can influence the number of sperm that reach the female SSTs, while the rate at which sperm egress from the SSTs is mostly determined by ejaculate quality [22,28]. A recent verbal model has proposed that ejaculate quality might modulate sperm release from the SSTs because sperm are flushed out from the SSTs when their swimming velocity drops below a threshold fluid current generated by glandular secretion in the distal end of the SST [29]. This hypothesis predicts a strong temporal pattern of competitive fertilisation. Slow-swimming sperm from a low-quality ejaculate are expected to leave the SSTs sooner than the fast-swimming sperm of a high quality-ejaculate. Therefore, controlling for sperm numbers, a low-quality ejaculate might have a high probability of fertilising the first eggs relative to the last eggs of a laying sequence produced by a female, while we expect the high-quality ejaculate to become more competitive over subsequent days [23,29]. However, these predictions have not been tested and the dynamics of sperm competition in internally fertilising species remain largely unresolved.

Domestic fowl offer a unique opportunity to study time-dependent sperm competition dynamics. First, established husbandry techniques enable the artificial insemination of ejaculates of known size and quality [23]. Second, hens are typically promiscuous and store viable sperm for a median period of approximately 14 days [23]. Third, hens ovulate daily [30]. Combined together, these factors present an ideal opportunity to study temporal dynamics of sperm competition by monitoring the paternity of embryos produced by a hen over successive days following the experimental insemination of competing ejaculates of controlled size and quality. In this study, we artificially inseminated a set of hens with engineered heterospermic ejaculates from two different males, one producing ejaculates of consistently low, and the other of consistently high quality, each insemination comprising either 40 × 10^6 ^sperm from the low-quality and 10 × 10^6 ^sperm from the high-quality male (4:1 treatment), or 20 × 10^6 ^from the low- and 10 × 10^6 ^from the high-quality male (2:1 treatment). By monitoring the paternity of the embryos produced over successive days following insemination, we were able to distinguish the independent effects of ejaculate size and quality on time-dependent variation in paternity.

## Methods

### Artificial insemination experiment

We measured sperm quality as sperm mobility, using an *in vitro *assay that measures the ability of a population of sperm to penetrate a solution of an inert medium (Accudenz: Accurate Chemicals & Scientific Corporation, Westbury, NY, USA) from an overlaid suspension. Sperm mobility was quantified as light absorbance units with a spectrophotometer [31,32]. The absorbance of a sperm population is proportional to the percentage of sperm that have a straight-line swimming velocity (VSL) greater than 30 μm/sec [32]. We studied a random-bred population of New Hampshire domestic fowl, characterised by males of highly repeatable sperm mobility, at Oregon State University, Corvallis (US) in 2004 [21,22,32,33]. We used 10 males all in their prime and of the same age (approximately 30 weeks [30]): 5 males producing sperm of high mobility (mean [± SE] 0.5771 ± 0.0234 abs. units) and 5 males of low sperm mobility (0.2460 ± 0.0187 abs. units). We constituted five pairs of males in which one male produced sperm of consistently and significantly higher mobility than the other (variation in sperm mobility across male pairs and between high and low mobility males within each male pair based on four semen samples collected from each male following sexual rest, male pair: F_4,39 _= 2.49, *p *= 0.064, male phenotype [nested within male pair]: F_5,39 _= 29.27, *p *< 0.0001). We selected 80 hens unrelated to the males, 40 from high-mobility families and 40 from low-mobility families (i.e. with high- or low-mobility full-sib brothers, respectively). We obtained a semen sample from both pair members and engineered a heterospermic ejaculate by mixing the sperm of the two males, through standard poultry techniques [21,34]. For each male pair, we subjected 40 hens (20 from high- and 20 from low-mobility families) to the 4:1 treatment, and the remaining 40 to the 2:1 treatment. Briefly, semen samples were collected from the two males of a pair through abdominal massage within 5 minutes of each other. Sperm numbers were measured in each sample through spectrophotometer readings and a standard curve. Hens were artificially inseminated with the engineered heterospermic ejaculate in quick succession, within 30 minutes of semen collection, and in random order with respect to mobility line. Following artificial insemination, hens were housed singly, eggs were collected daily and labelled by female and laying order. Embryos were collected for paternity assignment following 12 days of incubation. In the domestic fowl, embryo development requires 19–20 days of incubation [30]. By interrupting embryonic development on day 12 of incubation, we therefore reduced the risk that differential embryo mortality may bias the paternity results [34]. When they depleted the sperm stores from the artificial insemination of a male pair, as indicated by the consistent production of infertile eggs (> 14 days following insemination), individual hens were artificially inseminated with the heterospermic insemination of the same treatment (i.e. 2:1 or 4:1) from the next male pair. In other words, each hen was successively inseminated with the sperm of all five male pairs within the same treatment.

### Molecular methods

We extracted genomic DNA from approximately 1 μl of blood using a standard ammonium acetate procedure (modified from [35]). We genotyped all ten candidate male parents at an initial test set of 23 microsatellite loci. We then genotyped the maternal and progeny samples at a subset of four selected loci (*ADL0138*, *ADL0268 *[36]; *LEI0196 *[37]; *LEI0246 *[38]), chosen because they included alleles that would discriminate between paired experimental males. Chromosomal locations of each microsatellite were obtained using a BLASTN search on the ENSEMBL webpage http://www.ensembl.org/Gallus_gallus/blastview. The loci *ADL0268 *and *LEI0246 *are located on chromosome 1, and the loci *ADL0138 *and *LEI0196 *on chromosome 6. There was no evidence of linkage disequilibrium between any pair of loci, including those located on the same chromosomes, in the parental birds (*ADL0268 *&*LEI0246*; χ^2 ^= 3.06, df = 2, *p *= 0.22, *ADL0138 *&*LEI0196*; χ^2 ^= 3.82, df = 2, *p *= 0.15), and loci can therefore be treated as independent. DNA amplifications were performed in 10 μl reactions containing 10–50 ng of DNA, 80 μmol each primer, 0.16 mM dNTPs, 1.5 mM (*ADL0268*) or 2.0 mM (*ADL0138*, *LEI0196*, *LEI0246*) MgCl_2 _and 0.5 units *Taq *polymerase (Bioline). The PCR profile comprised an initial denaturation cycle of 5 minutes at 94°C followed by 35 cycles of 30 seconds each at 94°C, annealing temperature (52°C: *ADL0138 *&*LEI0246*; 58°C: *ADL0268 *&*LEI0196*), and 72°C, and terminated by a further 10-minute extension cycle at 72°C. PCR products were genotyped on an ABI 3730 DNA Analyzer and fragments analysed using GENEMAPPER software (Applied Biosystems).

### Parentage Analysis

In each of the five male pairs 2–3 of the four loci were diagnostic for paternity. Although a complete exclusion approach to paternity assignment is possible for this dataset, we used a likelihood-based approach implemented by CERVUS[39] to allow for any effects of genotyping error, assumed to be 0.01, which can lead to allelic mismatches between parents and progeny. All samples containing mismatches were re-amplified to verify genotypes. Females were included as known parents in CERVUS assignments, and the two potential fathers included as candidate parents. Paternity could be assigned with a confidence greater than 0.95 in all but 36 of the 1,928 progeny. Four further progeny could be assigned unambiguously by exclusion based on at least one locus, whereby progeny were required to share one allele per locus with the known mother, and the second allele with the putative father. Overall, we were therefore able to assign paternity to 1896/1928 progeny.

### Statistical Analysis

We analysed variation in the probability that the low mobility-male fathered young produced on each successive day of a laying sequence (i.e. day 1–12) averaged across all the eggs produced by all inseminated hens on each laying day, through a Linear Mixed Effects Model (LME) in R 2.7.1, with binomial error structure and logit link function, in which the proportion of young fathered by the low mobility-male of a pair on a given laying day was entered as the response variable, insemination treatment (i.e. 4:1 or 2:1) as a factor nested within male pair, laying order (i.e. day 1–12) and the ratio of sperm mobility of each male pair (i.e. mean sperm mobility of the low-mobility male/mean sperm mobility of the high-mobility male) as covariates, and male pair as a random effect [40]. In addition, we weighted each observation by the total number of offspring produced by each male pair on a given laying order [40], and considered two-, and three-way interactions between insemination treatment, laying order, and the mobility ratio of each male pair.

We also conducted a more parsimonious analysis by collapsing the dataset into a single mean probability of paternity by the low-mobility ejaculate across all five male pairs on each laying day (1–12) within each insemination treatment, through a Generalised Linear Model (GLM) in Minitab 15, in which mean probability of paternity by the low-mobility ejaculate was entered as the response variable, and laying order (1–12 in each treatment) and insemination treatment (2:1 and 4:1) were entered as covariates [41]. This analysis enabled us to test: (a) temporal effects on probability of paternity, and (b) insemination treatment-specific differences in the rate at which probability of paternity changes with time (laying order), by comparing slopes and intercepts of the regression functions in each treatment. A significant effect of insemination treatment would indicate that the intercepts of the two regressions are significantly different, while a significant insemination treatment × laying order interaction would indicate that two regression slopes are significantly different [41]. The collapsed dataset was normally distributed (Kolmogorov-Smirnov test = 0.162, *p *= 0.100).

## Results

**Figure 1:**
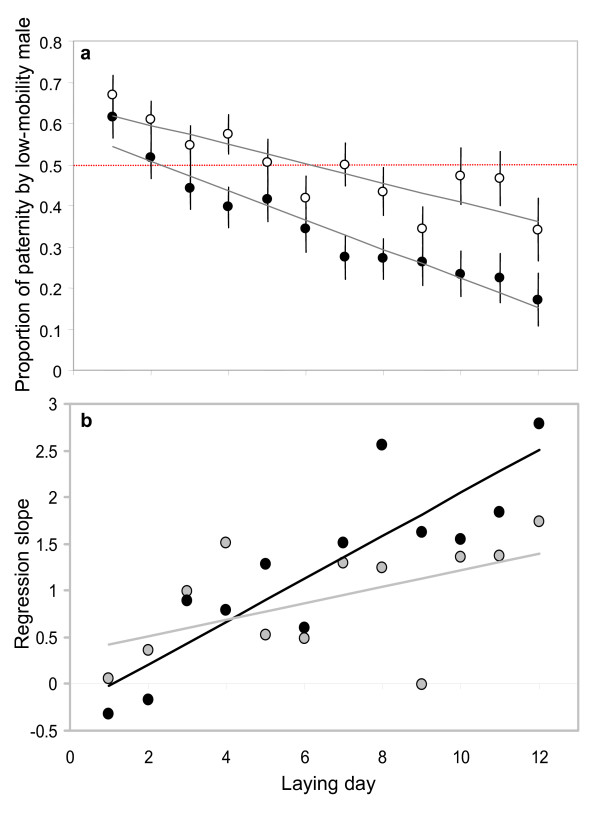
**Time-dependent dynamics of competitive fertilisation**. (a) The probability that a low-mobility ejaculate wins sperm competition declines drastically over a laying sequence, and more so when its numerical advantage is reduced. The fertilising advantage of the low-mobility ejaculates was restricted to the first eggs ovulated following insemination. The extent of this initial fertilising advantage was determined by the numerical superiority of the low-mobility ejaculate over the high mobility ejaculate. In the 4:1 treatment, the low-mobility ejaculate retained a fertilising advantage over the eggs produced in the first five days, in the 2:1 treatment, this fertilising advantage was restricted to the eggs produced in the first day. Data points represent paternity share averaged for all the hens of a male pair, and across the five male pairs (vertical bars: SE). (b) The difference in sperm mobility between competing ejaculates had a progressively stronger influence on paternity towards the last days of a laying sequence and more so when the numerical advantage to the low-mobility ejaculate was reduced (2:1 treatment). For each laying day (1–12), within each insemination treatment (2:1 and 4:1), we analysed the linear regression of the probability of paternity by the low-mobility ejaculate of a male pair over its mobility ratio (n = 5 for each treatment/laying day combination). The graph presents the slope (*b*) of these regression functions obtained over successive laying days for the 2:1 (black data points) and the 4:1 (grey) insemination treatments. The slope of probability of paternity over mobility ratio becomes steeper over the laying sequence, and more so in the 2:1 treatment.

There was a striking temporal pattern in variation in paternity share in both insemination treatments. Despite a non-significant overall tendency for the low-mobility ejaculate to fertilise more eggs when it outnumbered the high-mobility ejaculate 4:1 rather than 2:1 (LME, treatment: t_1,3 _= 1.449, effect estimate ± SE = 0.295 ± 0.203, *p *= 0.243, Figure 1a), the probability of fertilisation by the low-mobility ejaculate declined linearly over successive days following insemination (LME, laying order: t_1,106 _= -3.922, effect estimate = -0.073 ± 0.019, *p *= 0.0001; Figure 1a). Consistent with these findings, the mean probability of paternity by the low-mobility ejaculate changed significantly over time (i.e. laying order) within each individual male pair in both treatments, and in four out of the five male pairs, the probability of paternity by the low-mobility ejaculate declined more sharply in the 2:1 treatment than in the 4:1 treatment (median algebraic difference in regression slope across the five male pairs, 2:1 – 4:1 treatment = -0.029).

Despite a strong numerical advantage, low-mobility ejaculates failed to out-compete high-mobility ejaculates. In the 2:1 treatment, we would expect the low-mobility ejaculate to fertilise two thirds of the eggs, however this rate was only approached for the eggs produced on the first day following an insemination (1-sample T test, t = -1.13, *p *= 0.32, n_male pairs _= 5), with the proportion of eggs fertilised by the high-mobility ejaculate increasing progressively on subsequent days. In the 4:1 treatment, the low-mobility ejaculate failed to fertilise 80% of the eggs on the first day (t = -3.47, *p *= 0.026), and – again – its fertilising advantage further declined over successive days. Therefore, our results suggest that temporal patterns in fertilisation success determined by variation in sperm mobility might be more pronounced when the numerical advantage to the low-mobility ejaculate is relatively low (i.e. 2:1 treatment), as reflected by a non-significant tendency for an interaction between insemination treatment and laying order (LME, t_1,106 _= -1.753, effect estimate = -0.0459 ± 0.026, *p *= 0.0825). The effect of sperm mobility was further confirmed by the fact that the temporal decline in fertilising efficiency of the low-mobility ejaculate was reduced in inseminations in which the difference in sperm mobility between the competing ejaculates (i.e. the ratio of low to high mobility was high) was relatively low (LME, mobility ratio × laying order: t_1,106 _= 2.108, effect estimate = 0.090 ± 0.043, *p *= 0.0374). This temporal effect of relative sperm mobility was particularly marked in the 2:1 insemination treatment, where the numerical advantage to the low-mobility ejaculate was limited (LME, insemination treatment × laying order × mobility ratio: t_1,106 _= 2.334, effect estimate = 0.140 ± 0.060, *p *= 0.0215, Figure 1b).

The analysis of mean probability of paternity by the low-mobility ejaculate across all five male pairs confirmed a significant decline with laying order (GLM, F_1,20 _= 74.36, *p *< 0.0001, adj. R^2 ^= 86.57%), and while this analysis also failed to detect any effect of insemination treatment (GLM, F_1,20 _= 1.38 *p *= 0.253), there was a significant interaction between insemination treatment and laying order (GLM, F_1,20 _= 5.82, p = 0.026). Taken together, these results indicate that: (a) the probability that a low-mobility ejaculate wins sperm competition declines over time, and (b) this decline is slower when the low-mobility ejaculate outnumbers the high-mobility ejaculate 4:1 rather than 2:1.

## Discussion and conclusion

These results reveal a striking and previously undetected, time-dependent effect of sperm mobility which leads low-mobility ejaculates to lose their fertilising ability at a faster rate than high-mobility ejaculates. We show that shortly following competitive insemination, ejaculate size is an important predictor of paternity, but with prolonged sperm storage, ejaculate quality becomes an over-riding factor. This is the first demonstration of the patterns of sperm competition dynamics within the SSTs in birds. A recent model proposes that sperm are flushed out from the SSTs when their swimming velocity drops below a threshold fluid current generated by glandular secretion in the distal end of the SST [23]. Because low-mobility ejaculates contain a higher proportion of slow-swimming sperm, this model predicts that low-mobility ejaculates will exit the SSTs before high-mobility ejaculates. The time-dependent sperm competition dynamics revealed in this study, are consistent with this prediction. Low-mobility ejaculates may suffer higher rates of post-meiotic sperm senescence [19,20]. Recent work on this study population would suggest that both higher rates of egression of live sperm from the SSTs and faster rates of sperm necrosis [28,33] may account for the strong temporal effect detected in probability of fertilisation by low-mobility ejaculates. It is indeed possible that both mechanisms may represent integrated parts of the same senescence pathway because swimming velocity in birds may decline as sperm age [33,42]. The observation that in the 2:1 treatment low mobility ejaculates only just achieved the expected fertilisation success on the first day, and failed to achieve this even on the first day in the 4:1 treatment, strongly suggests that, in addition to the rate of egression from the SSTs, sperm mobility may also be important in determining the number of live sperm that are initially stored within the female SSTs, as suggested by previous work [22,28]. This pattern may be explained by the fact that low-mobility ejaculates contain a higher proportion of non-motile sperm which cannot be stored within the SSTs [28,33].

Regardless of the specific underlying mechanisms, these time-dependent sperm competition dynamics have important repercussions for the evolution of male reproductive strategies. First, sperm competition theory predicts that males from populations, or from individual genotypes within a population, that experience consistently high levels of sperm competition should produce sperm at a faster rate [43,44]. While there is comparative and experimental evidence consistent with this expectation [reviewed in [45]], recent work indicates that in addition to the number of sperm inseminated, the motile performance of sperm can also play a critical role in sperm competition [14-16,42]. The results of the present study are entirely consistent with this, and indicate that variation in sperm quality can over-ride even substantial variation in sperm numbers, particularly over prolonged periods of sperm storage. Therefore, our study suggests that an increment in the metabolic performance of the sperm produced may be an efficient – and often neglected – evolutionary response to sperm competition that can complement or supplement an increase in sperm numbers. This response might be particularly relevant in species with prolonged female sperm storage.

Second, the results of the present study indicate that – for a given amount of sperm available – males producing low quality ejaculates may be selected to inseminate a female with smaller ejaculates repeatedly over successive days, rather than inseminate her with a single large ejaculate. One way to achieve this would be for males to monopolise sexual access to individual females. Consistent with this, a negative relationship appears to occur in the fowl between social status and sperm mobility both across [22] and within individual males [46,47]. More generally, the present results might help explain why in some species, males with relatively low sperm quality invest preferentially in social competitive ability [e.g. [48]].

In conclusion, the results of the present study provide an experimental demonstration of the mechanisms of sperm competition during prolonged female sperm storage, and by revealing marked time-dependent dynamics determined by variation in sperm quality, shed light on the evolution of different male reproductive strategies across and within populations. However, our study was restricted to a well characterised study population and based on a limited sample size. Future work should explore the generality of these results. For example, it would be particularly important to establish how the impact of differential rates of sperm senescence on sperm competition changes with varying duration of female sperm storage across a range of species.

## Authors' contributions

TP conceived the study, wrote the research grant to fund the work, analysed the results, and wrote the paper with the other co-authors. DPF developed the mobility assay, managed the study population, and conducted the experiments with TP. KW conducted the molecular work and paternity analysis under TB's supervision.
